# Community Transmission of SARS-CoV-2 at Three Fitness Facilities — Hawaii, June–July 2020

**DOI:** 10.15585/mmwr.mm7009e1

**Published:** 2021-03-05

**Authors:** Laura M. Groves, Lauren Usagawa, Joe Elm, Eleanor Low, Augustina Manuzak, Joshua Quint, Katherine E. Center, Ann M. Buff, Sarah K. Kemble

**Affiliations:** ^1^University of Hawaii, Honolulu, Hawaii; ^2^Hawaii Department of Health; ^3^CDC COVID-19 Response Team.

On July 2, 2020, the Hawaii Department of Health was notified that a fitness instructor (instructor A) had experienced signs and symptoms compatible with coronavirus disease 2019 (COVID-19)* and received a positive reverse transcription–polymerase chain reaction (RT-PCR) test result for SARS-CoV-2, the virus that causes COVID-19. At the time, Honolulu County reported community transmission of a 7-day average of 2–3 cases per 100,000 persons per day (*1*). Before the onset of symptoms, instructor A taught classes at two fitness facilities in Honolulu, facilities X and Y. Twenty-one COVID-19 cases were linked to instructor A, including a case in another fitness instructor (instructor B). The aggregate attack rates in classes taught by both instructors <1 day, 1 to <2 days, and ≥2 days before symptom onset were 95% (20 of 21), 13% (one of eight), and 0% (zero of 33), respectively. Among the 21 secondary cases, 20 (95%) persons had symptomatic illness, two (10%) of whom were hospitalized. At the time of this outbreak, use of masks was not required in fitness facilities. To reduce SARS-CoV-2 transmission in fitness facilities, staff members and patrons should wear a mask (including during high-intensity exercise), and facilities should implement engineering and administrative controls including 1) improving ventilation; 2) enforcing consistent and correct mask use and physical distancing (maintaining ≥6 ft of distance between all persons, limiting physical contact and class size, and preventing crowded spaces); 3) reminding all patrons and staff members to stay home when ill; and 4) increasing opportunities for hand hygiene. Conducting exercise activities entirely outdoors or virtually could further reduce SARS-CoV-2 transmission risk.

## Investigation and Results

The Hawaii Department of Health conducted a cluster investigation across three fitness facilities. The index patient, fitness class participants, and facility staff members were interviewed using a standardized questionnaire; clinical and SARS-CoV-2 molecular test records were reviewed; and on-site facility assessments were conducted. This activity was reviewed by CDC and was conducted consistent with applicable federal law and CDC policy.^†^

The index case occurred in a male fitness instructor (instructor A) aged 37 years. Instructor A reported onset of fatigue on the evening of June 29 (Figure). The next day, he reported chills, body aches, cough, congestion, sore throat, and headache. On July 1, he received a positive SARS-CoV-2 RT-PCR test result.

**FIGURE:**
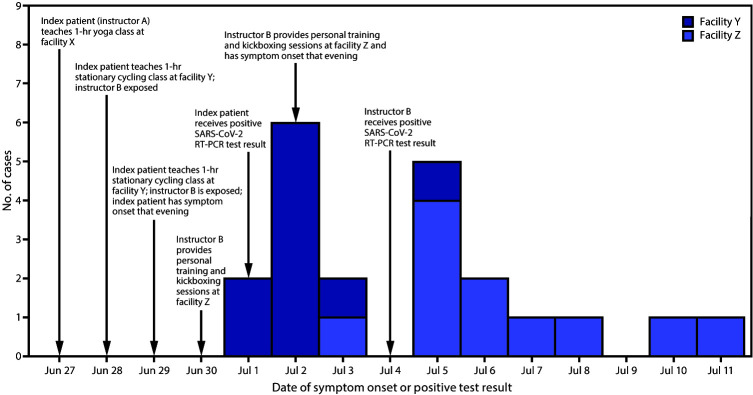
Date of symptom onset or positive test result* among 21 COVID-19 cases epidemiologically linked to a fitness center instructor — Hawaii, June 29–July 11, 2020 **Abbreviations:** COVID-19 = coronavirus disease 2019; RT-PCR = reverse transcription–polymerase chain reaction. * Date of positive SARS-CoV-2 test result was used for one asymptomatic patient from facility Z.

Before his symptoms began, instructor A taught classes at two fitness facilities (facilities X and Y) on June 27, 28, and 29 (Table 1). On the morning of June 27, >2 days (60 hours) before symptom onset, instructor A taught a 1-hour yoga class for 27 participants at facility X. Instructor A wore a mask, but no participants wore masks. No participants reported symptoms during the next 14 days.^§^ Only one (4%) participant received SARS-CoV-2 RT-PCR testing, with a negative result, on July 3. Thus, the attack rate for facility X on June 27 was 0% (zero of 27) (Table 2).

**TABLE 1:** Timeline of classes and characteristics of instructors and participants at three fitness facilities — Hawaii, June 27–July 11, 2020

Fitness facility	Date	Class time	Exercise	Instructor	Instructor masked	No. of participants (no. masked)	No. of participants with SARS-CoV-2 test and outcome*			Attack rate, %^§^
							RT-PCR positive	RT-PCR negative	Asymptomatic, not tested^†^	
X	Jun 27	AM	Yoga	A	Yes	27 (0)	0	1	26	0
Y	Jun 28	PM	Stationary cycling	A	No	4 (0)^¶,^**	0	4	0	0
Y	Jun 29	PM	Stationary cycling	A	No	10 (0)**	10	0	0	100
Z	Jun 30	AM	PT	B	Yes	3 (3)^¶^	0	1	2	0
Z	Jun 30	PM	Kickbox	B	No	3 (0)^¶^	0	0	3	0
Z	Jul 1	AM	PT	B	No	4 (0)	1	2	1	25
Z	Jul 2	AM	PT	B	No	2 (0)^††^	1	1	0	50
Z	Jul 2	PM	Kickbox	B	No	9 (2)	9	0	0	100

**Abbreviations:** PT = personal training; RT-PCR = reverse transcription–polymerase chain reaction.

* SARS-CoV-2 RT-PCR test result conducted within 2 weeks of last exposure.

^†^ Participant was not tested for SARS-CoV-2 and reported no fever, cough, shortness of breath, muscle or body aches, fatigue, headache, congestion, runny nose, sore throat, or new loss of sense of taste or smell for 14 days after last exposure.

^§^ Participants who were not tested and reported no symptoms for 14 days after exposure were considered not infected and categorized as negative for attack rate calculations.

^¶^ Participants who had more than one exposure date to the instructor were only included in the class counts and attack rate for the last exposure date.

** Instructor B was a class participant.

^††^ Included a caregiver of a participant.

**TABLE 2:** COVID-19 attack rates* among participants in classes conducted by instructors A and B who taught at three fitness facilities while infected with SARS-CoV-2, by number of days before instructor symptom onset — Hawaii, June 29–July 11, 2020

Days before symptom onset	Instructor A^†^			Instructor B^†^			Instructors A and B^§^	
	Instructor masked	No. of class participants exposed (no. masked)	No. of cases (attack rate)	Instructor masked	No. of class participants exposed (no. masked)	No. of cases (attack rate)	No. of class participants	No. of cases (attack rate, %)
≥2	Yes	27 (0)	0 (—)	AM: Yes PM: No	6 (3)	0 (—)	33	0 (—)
<2 to 1	No	4 (0)	0 (—)	No	4 (0)	1 (25)	8	1 (13)
<1	No	10 (0)	10 (100)	No	11 (2)	10 (91)	21	20 (95)

**Abbreviation:** COVID-19 = coronavirus disease 2019.

* Percentage of cases among all participants.

^†^ Instructor B is included among instructor A’s class participants.

^§^ No participants were exposed to both instructors.

On June 28, less than 2 but more than 1 day (38 hours) before symptom onset, instructor A taught a 1-hour high-intensity stationary cycling class for 10 participants at a second facility, facility Y. Instructor A and participants followed facility Y’s protocol and did not wear masks during exercise (*2*). The stationary cycling room measured 24 ft by 17 ft (408 sq ft). Doors and windows were closed, and three large floor fans were directed toward the participants for cooling. Instructor A was on a pedestal facing participants, shouting instructions and encouragement. Instructor A was >6 ft away from participants, and cycling stations were ≥6 ft apart. Among 10 participants, four had exposure to instructor A only during this class; all four received negative SARS-CoV-2 RT-PCR test results during July 3–4 (attack rate = 0% [zero of four]). Six participants had additional exposure to instructor A the next day.

On June 29, 4 hours before symptom onset, instructor A taught a 1-hour stationary cycling class with 10 participants at facility Y in the same format and room as that on June 28 (Table 1). No one wore masks while exercising. Six of the participants were those who had exposure to instructor A the previous day, and four participants had no exposure the day before. All 10 participants received positive SARS-CoV-2 RT-PCR test results during July 2–6 (attack rate = 100% [10 of 10]) (Table 2). Among these 10 cases at facility Y, seven occurred in women, seven patients identified as Asian and three as Native Hawaiian or Pacific Islander, and their median age was 37 years (range = 31–50 years). All patients were symptomatic, one of whom was another fitness instructor, instructor B.

Instructor B, a man aged 46 years, worked as a personal trainer at a third facility, facility Z. On the evening of July 2, 4 days after his first exposure to instructor A, instructor B reported body aches and sore throat with progression of symptoms, including fever, chills, cough, shortness of breath, and fatigue. On July 4, instructor B received a positive RT-PCR test result for SARS-CoV-2; he was later hospitalized and required admission to an intensive care unit.

On June 30, 2 days after first exposure at facility Y and ≥2 days before his symptom onset, instructor B taught five personal training and small-group kickboxing sessions with 10 participants and a participant caregiver at facility Z. For six participants (three in the morning and three in the afternoon), June 30 was their only exposure to instructor B. Sessions were 1 hour in duration, and physical distancing was not maintained except by the caregiver. Everyone in morning personal training sessions, including instructor B, wore a mask. No one in the afternoon kickboxing sessions, including instructor B, wore a mask. One of these participants received a negative SARS-CoV-2 molecular test result on July 6; five reported no symptoms during the next 14 days (attack rate = 0% [zero of six]) (Table 2). Four participants and the caregiver had additional exposure to instructor B on July 2.

On July 1, less than 2 but more than 1 day (36 hours) before symptom onset, instructor B provided personal training to four different participants at facility Z. No one wore masks. Among these four participants, one reported no symptoms in the next 14 days, and two received negative test results (one on July 6; one on both July 8 and July 16), and one received a positive SARS-CoV-2 RT-PCR test result on July 6 (attack rate = 25% [one of four]).

On July 2, 12 hours before symptom onset, instructor B taught 10 participants (one personal training and three kickboxing sessions with nine participants) with the caregiver present (11 exposed persons); four participants and the caregiver also participated in sessions on June 30. This was the only exposure to instructor B for the other six persons. Instructor B did not wear a mask. Two participants wore masks; both were infected. One had exposure on both June 30 and July 2, and one had exposure only on July 2. All received SARS-CoV-2 RT-PCR testing, and nine participants and the caregiver received positive results during July 6–8. The attack rate was 91% (10 of 11).

Among 11 cases from facility Z, seven were in women, seven patients identified as Asian, three as White, and one as Native Hawaiian or Pacific Islander, and their median age was 61 years (range = 53–81 years). Two (18%) participants had Parkinson disease. Ten patients were symptomatic, and one was hospitalized and required admission to an intensive care unit. The aggregate attack rates for both instructors by timing of exposure relative to their symptom onset dates were 0% (zero of 33), 13% (one of eight), and 95% (20 of 21), for exposure ≥2 days, <2 days to 1 day, and <1 day before symptom onset respectively (Table 2).

## Public Health Response

The Hawaii Department of Health provided isolation and quarantine instructions to all participants with exposure to instructors A and B in facilities Y and Z. Facility X participants were not quarantined because their exposure was >48 hours before instructor A’s symptom onset. In response to increasing COVID-19 case rates and fitness facility clusters, Honolulu City and County amended emergency orders on July 22, 2020, to require that all persons wear face coverings (i.e., nonmedical masks) in fitness facilities, including during exercise (*3*).

Facility Y installed plexiglass barriers between stationary cycling cycles, removed four cycles from the stationary cycling room, limited classes to six participants, and instituted facility-wide single-direction foot traffic flow. All three facilities began checking patrons’ temperatures upon entry; facility Y also required signed affirmations that patrons did not have COVID-19–compatible symptoms.

## Discussion

In this SARS-CoV-2 cluster investigation among fitness class participants exposed to fitness instructors who taught before their symptom onset, but while potentially infectious, the rate of transmission was highest on the day of symptom onset for both instructors, which is consistent with findings from a previous study; persons infected with SARS-CoV-2 are most infectious from 2 days before to 7 days after symptom onset (*4*). Transmission was likely facilitated by not wearing face masks, extended close contact, and poor room ventilation. SARS-CoV-2 transmission occurred despite stationary cycles being spaced ≥6 ft apart. Instructor A’s shouting throughout the 1-hour stationary cycling class might have contributed to transmission; aerosol emission during speech has been correlated with loudness (*5*), and COVID-19 outbreaks related to intense physical activity and singing have been previously reported (*6*–*8*).

This COVID-19 cluster occurred when SARS-CoV-2 community transmission was low (daily average of 2–3 cases per 100,000) (*1*). To reduce SARS-CoV-2 transmission in fitness facilities, staff members and patrons should wear a mask, and facilities should combine engineering and administrative controls including improving ventilation; enforcing consistent and correct mask use and physical distancing (maintaining ≥6 ft of distance between all persons, limiting physical contact and class size, and preventing crowded spaces); increasing opportunities for hand hygiene; and reminding patrons and staff members to stay home when ill. Conducting exercise activities entirely outdoors or virtually could further reduce SARS-CoV-2 transmission risk. As of February 2021, CDC guidance for fitness facilities recommends using the occupational hazard hierarchy of controls and combining controls to prevent SARS-CoV-2 transmission (*9*). Facilities should increase or improve ventilation by maximizing fresh air delivered to occupied spaces; increasing filter efficiency of heating, ventilation, and air conditioning units; using portable high-efficiency particulate air filtration units where indicated; and ensuring that fans do not direct air from one patron to another (*9*). Additional engineering and administrative controls include modifying fitness areas to provide ≥6 ft of physical distance between patrons, installing physical barriers, making foot traffic flow in a single direction, using visual cues for physical distancing, and adding hand sanitizer stations (*9*). Adding multiple engineering and administrative controls, including enforcing consistent and correct mask use for staff members and patrons, cleaning with Environmental Protection Agency–registered products for surface disinfection, and reducing facility occupancy and class sizes, are recommended to further reduce transmission risk (*9*).

The findings in this report are subject to at least three limitations. First, many participants had multiple dates of exposure; attack rate was calculated based on participants’ most recent exposure day, although exposure effects might have been cumulative. Second, the true number of participants infected with SARS-CoV-2 might have been underestimated. Many asymptomatic participants did not receive SARS-CoV-2 tests because of personal reluctance or lack of available testing. Finally, participants might have underreported symptoms or refused testing because of recall bias or social desirability bias or to avoid isolation.

This cluster investigation highlights the high transmissibility of SARS-CoV-2 in certain settings, including indoor fitness facilities. To reduce SARS-CoV-2 transmission in fitness facilities, staff members and patrons should wear a mask, and facilities should implement engineering and administrative controls including improving ventilation, enforcing physical distancing and consistent and correct mask use (even during high-intensity activities)^¶^ (*10*), increasing opportunities for hand hygiene, and reminding all patrons and staff members to stay home when ill. Conducting exercise activities entirely outdoors or virtually could further reduce SARS-CoV-2 transmission risk.

**Summary**

**What is already known about this topic?**

COVID-19 outbreaks have been reported from fitness and sports facilities.

**What is added by this report?**

Twenty-one COVID-19 cases were linked to an index case in a fitness instructor, who, along with a patient who was also an instructor, taught classes <1 day, 1 to <2 days, and ≥2 days before symptom onset; aggregate attack rates were 95%, 13%, and 0%, respectively.

**What are the implications for public health practice?**

To reduce SARS-CoV-2 transmission in fitness facilities, staff members and patrons should wear a mask, and facilities should enforce consistent and correct mask use (including during high-intensity activities) and physical distancing, improve ventilation, and remind patrons and staff members to stay home when ill. Exercising outdoors or virtually could further reduce SARS-CoV-2 transmission risk.

## Footnotes

* Signs and symptoms included fever, cough, shortness of breath, muscle or body aches, fatigue, headache, congestion or runny nose, sore throat, and new loss of sense of taste or smell.

† 45 C.F.R. part 46, 21 C.F.R. part 56; 42 U.S.C. Sect. 241(d); 5 U.S.C. Sect. 552a; 44 U.S.C. Sect. 3501 et seq. Participants provided oral consent for interviews and clinical record reviews.

§ Participants who were not tested and reported no symptoms for 14 days after exposure were considered not infected and categorized as negative for attack rate calculations.
